# Hydrogen bridges of polycyclic aromatic systems with O-H···O bonds — a gas-phase vs. solid-state Car-Parrinello study

**DOI:** 10.1007/s00894-014-2550-8

**Published:** 2015-01-24

**Authors:** Jarosław J. Panek, Aneta Jezierska

**Affiliations:** Faculty of Chemistry, University of Wrocław, F. Joliot-Curie 14, 50-383 Wrocław, Poland

**Keywords:** Car-Parrinello molecular dynamics, Intramolecular O-H···O hydrogen bond, Naphthol derivatives, Vibrational features

## Abstract

The current study belongs to a series of investigations of polycyclic aromatic compounds containing intramolecular hydrogen bonds. Close proximity of the coupled aromatic system and hydrogen bridges gives rise to resonance-assisted hydrogen bonding phenomena. Substituted naphthols are ideally suited for this kind of investigation. The parent compound, 1-hydroxy-8-methoxy-3-methylnaphthalene, and its derivative, 1-bromo-5-hydroxy-4-isopropoxy-7-methylnaphthalene, both with known crystal structure, are investigated. Car-Parrinello molecular dynamics (CPMD) is chosen as a theoretical background for this study. Gas phase and solid state simulations are carried out. The effect of Grimme’s dispersion corrections is also included. The report presents time evolution of structural parameters, spectroscopic signatures based on the CPMD simulations, and comparison with available experimental data. We show that the proton transfer phenomena do not occur within the simulations, which is consistent with evaluation based on the acidity of the donor and acceptor sites. The effects of the substitution in the aromatic system and change of the environment (gas vs. condensed phase) are of similar magnitude.

## Introduction

**Fig. 1:**
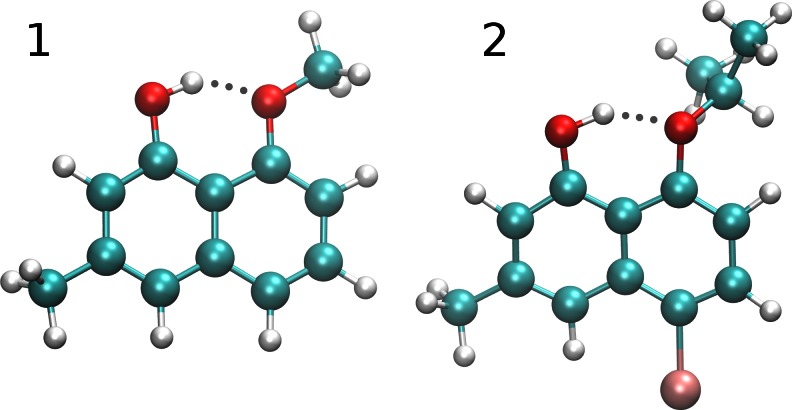
The molecular structures of 1-hydroxy-8-methoxy-3-methylnaphthalene (*1*) — left and 1-bromo-5-hydroxy-4-isopropoxy-7-methylnaphthalene (*2*) — right. The dotted line indicates the presence of the intramolecular hydrogen bond

**Scheme 1:**
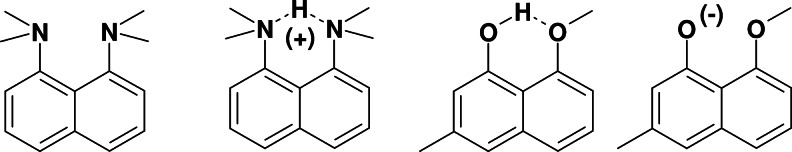
Molecular structures of 1,8-bis(dimethylamino)naphthalene (DMAN); its protonated form DMANH^+^, and 1-hydroxy-8-methoxy-3-methylnaphthalene in neutral form and as a phenolate

Hydrogen bonds are objects of experimental and theoretical studies due to their importance and diversity [1, 2]. In the current paper, two naphthalene derivatives containing O-H···O intramolecular hydrogen bonds (1-hydroxy-8-methoxy-3-methylnaphthalene (**1**) [3], and 1-bromo-5-hydroxy-4-isopropoxy-7-methylnaphthalene (**2**) [4]) are investigated on the basis of first-principle molecular dynamics based on Car-Parrinello scheme [5]. The study is a continuation of our investigations on hydrogen bonds in various systems, e.g., refs. [6–9]. The studied polycyclic aromatic compounds contain resonance-assisted hydrogen bonds, in which donor and acceptor moieties are located on neighboring fused rings [10]. Quasi-rings are formed due to the presence of the intramolecular hydrogen bonds (see Fig. 1). The oxygen atoms are bonded to carbon atoms in positions 1 and 8 of the naphthalene skeleton, and this central location of the hydrogen bonding yields balanced possibilities of modification by introduction of substituents. Thus, inductive and steric effects easily influence the hydrogen bonding strength and its dynamics. Knowing that the proton transfer phenomena cannot occur in the studied cases, the investigations were designed to study the hydrogen bridge dynamics and substitution related effects. As shown in Fig. 1, the compound denoted as **1** possesses only a methyl group as a substituent in the aromatic moiety. As such, it is a good reference structure to show the contribution of substituents to the bridge dynamics. The compound **2** is substituted in two positions, and its intramolecular hydrogen bonding is modulated by the inductive and steric effects. At this point it is worth mentioning the naphthalene derivatives which gave impulse for the current study; these derivatives, called “proton sponges” (see Scheme 1), contain N-H···N hydrogen bond. 1,8-Bis(dimethylamino)naphthalene (DMAN) and its protonated form (DMANH^+^) are well-established in the literature [11] due to their easily modified molecular features, and exhibit very low barrier for proton transfer [12]. This group of compounds has been a subject of several reviews [13, 14] and continues to bring new structural possibilities of modification [15]. The conformation of DMAN is determined by interplay of several effects. Among them, the steric inhibition of resonance, van der Waals repulsions and dipole-dipole repulsions are classified as the most important. In addition, DMAN is flexible, which is associated with the presence of the NMe_2_ groups. The dynamics of the NMe_2_ groups involves rotations around N-C_ar_, in-plane or out-of-plane movements where the distance between nitrogen atoms is modified [16]. The objects of this study, compounds **1** and **2**, cannot be classified as “proton sponges”, but they exhibit structural similarities to that group related to flexibility of the system and distance modulation between the donor and acceptor atoms. The free rotations of the OH group from one side and methoxy or isopropoxy groups from the other side are restricted because of the stabilizing intramolecular hydrogen bond. Moreover, both hydrogen bonds, these present in DMANH^+^ and in the studied cases, are resonance-assisted, but — as explained in Scheme 1 — the “proton sponge”-like behavior could be assigned to the phenolate anions of **1** and **2**, not to the compounds themselves. Indeed, the pK_a_’s of naphthols are comparable to phenols (pK_a_ of phenol and 1-naphthol is 9.9 and 9.3 respectively), and the equilibrium is shifted toward the neutral, phenolic forms. The study will therefore concentrate rather on the impact of substitution on the molecular properties.

Car-Parrinello molecular dynamics was employed as the main theoretical approach to provide data on time-evolution of the metric parameters. The obtained trajectories served further in the Fourier transforms of atomic velocities to shed light onto spectroscopic features of both studied compounds. All computations were performed with Grimme’s dispersion correction [17], so that an as full as possible set of strong and weak inter-and intramolecular interactions could be included during the simulations. The study is carried out for two phases, gas and solid, to enable the description of environmental effects related to the crystalline phase. Summarizing, the main aim of the study is a detailed investigation of the proton dynamics with special attention paid to the spectroscopic signatures as main detectors of the presence of substituents and their influence on the OH bond properties. The organization of the article is as follows: computational details are given in computational methods, while results and discussion are presented in the ensuing section; conclusions are given in the last section.

## Methods

Car-Parrinello molecular dynamics (CPMD) [5] was applied to develop time-evolution models for the studied compounds, 1-hydroxy-8-methoxy-3-methylnaphthalene (**1**), and 1-bromo-5-hydroxy-4-isopropoxy-7-methylnaphthalene (**2**). The structures of the compounds are presented in Fig. 1. The simulations were designed to represent gas and condensed phases for the studied compounds. The experimental X-ray data of the unit cells [3, 4], measured at 293 K, were used: for **1**, monoclinic, *a* = 14.163 Å, *b* = 5.009 Å, *c* = 14.869 Å, β = 113.61°, *Z* = 4; for **2**, monoclinic, *a* = 9.655 Å, *b* = 14.337 Å, *c* = 9.692 Å, β = 108.07°, *Z* = 4. The solid state simulations were performed with periodic boundary conditions (PBCs) and with real-space electrostatic summations for the eight nearest neighbors in each direction (TESR = 8). For the gas phase computations, the single molecules of **1** and **2** were placed in cubic cells with *a* = 18 Å for **1** and 20 Å for **2** respectively; the Hockney Poisson solver [18] was used to remove periodic images of the cell. The translational and rotational movements were continually removed during the gas phase computations to ensure that the molecule does not drift from the center of the cell. The Perdew, Burke, Ernzerhof (PBE) functional [19] and Troullier-Martins pseudopotentials [20] were applied throughout the study to reproduce the electronic structure. The plane wave kinetic energy cutoff value for the gas phase and solid state models was set at 100 Ry. The fictitious electron mass parameter was set to 400 a.u., while the CPMD equations propagation time step was set to 3 a.u. The simulations were performed at *T* = 297 K; to control the assumed temperature a Nosé-Hoover thermostat chain was applied [21, 22]. The empirical dispersion correction during the MD was computed according to Grimme [17]. The initial part of the MD runs was considered as equilibration time; thus, the first 10,000 steps were not considered in further analyses. The collected trajectories served to analyze structural and vibrational spectroscopic properties of the studied compounds in vacuo and in the solid state. The spectroscopic signatures were obtained via Fourier transforms of the atomic velocities to produce the relevant power spectra. The simulations were performed using the CPMD program v3.15.3 [23]. The data was analyzed with the VMD 1.8.6 [24] and Gnuplot [25] programs.

## Results and discussion

As we mentioned above, the motivation for this work is not investigation of the intramolecular proton transfer phenomena. They are unlikely to occur because of the chemical environment of the phenolic proton, i.e., the difference of acidities (or proton affinities) of the donor and acceptor sites. Instead, the main motivation was gaining insight into the impact of substituent effects on the structural behavior of naphthol derivatives. The framework of the investigated compounds is rigid and additionally stabilized by the presence of the quasi-ring of the resonance-assisted hydrogen bond. However, the use of molecular dynamics allows us to observe the effect of introduction of bromine and isopropyl functions (inductive and steric effects) on the dynamics of the bridge proton, including its tendency to deviate from the molecular plane. The possibility of this is compounded by the heavy isopropyl substituent at the acceptor side in **2**, which increases the local moment of inertia. Molecular dynamics schemes are well suited for this kind of investigation, because they allow focusing on the selected degrees of freedom while including the remaining degrees of freedom in a statistically averaged manner. The investigated compounds belong to the class rich in possible π-π interactions, therefore we include the empirical corrections for dispersion. This is especially valuable in the solid state.

Description of the substituent effects has been traditionally performed by the use of additive substituent constants, of which a large variety is available [26]. Such constants can also be used in the investigations of substituent-modified hydrogen bonding in the aromatic molecules [27]. The methyl substituent, present in **1**, is virtually neutral with respect to the inductive effect (Swain-Lupton constant *F* = 0.01) and slightly electron-donating by resonance (*R* = −0.18). On the other hand, the bromine atom present in **2** is inductively electron-withdrawing (*F* = 0.45), which is partially compensated by its resonance-mediated electron-donating properties (*R* = −0.22) [26]. The hydrogen bond acceptors are very similar in their inductive effect (*F*(OMe): 0.29, *F*(OCHMe_2_) = 0.34), while — according to Table 1 of ref. [26] — the resonance effect for the OCHMe_2_ group is not accurately determined. These data will be used below to illustrate the observed substituent effects.

**Table 1:** Metric parameters of the intramolecular hydrogen bonds of **1** and **2**: experimental (refs. [3, 4]) and computational — averages and standard deviations taken from CPMD runs in the solid state and gas phase

Metric parameters	O···O [Å]	O-H [Å]	H···O [Å]	OHO [°]	CCOH [°]
1-hydroxy-8-methoxy-3-methylnaphthalene (**1**)					
Experimental [3]	2.581(3)	0.843(30)	1.826(30)	148(3)	−4(2)
CPMD (solid state)	2.615 ± 0.080	0.989 ± 0.026	1.757 ± 0.111	143.49 ± 6.51	6.40 ± 10.01
CPMD (gas phase)	2.634 ± 0.090	0.985 ± 0.026	1.785 ± 0.121	143.11 ± 6.93	−0.49 ± 12.14
1-bromo-5-hydroxy-4-isopropoxy-7-methylnaphthalene (**2**)					
Experimental [4]	2.563(5)	0.894(40)	1.763(40)	148(4)	5(3)
CPMD (solid state)	2.584 ± 0.079	0.990 ± 0.027	1.731 ± 0.112	142.48 ± 6.85	2.98 ± 10.72
CPMD (gas phase)	2.615 ± 0.083	0.989 ± 0.027	1.747 ± 0.110	145.04 ± 6.70	−0.04 ± 11.51

The crystal structures were determined with the following accuracy: (**1**) wR(F) = 0.069 [3]; (**2**) wR(F) = 0.052 [4]

### Structural parameters

**Fig. 2:**
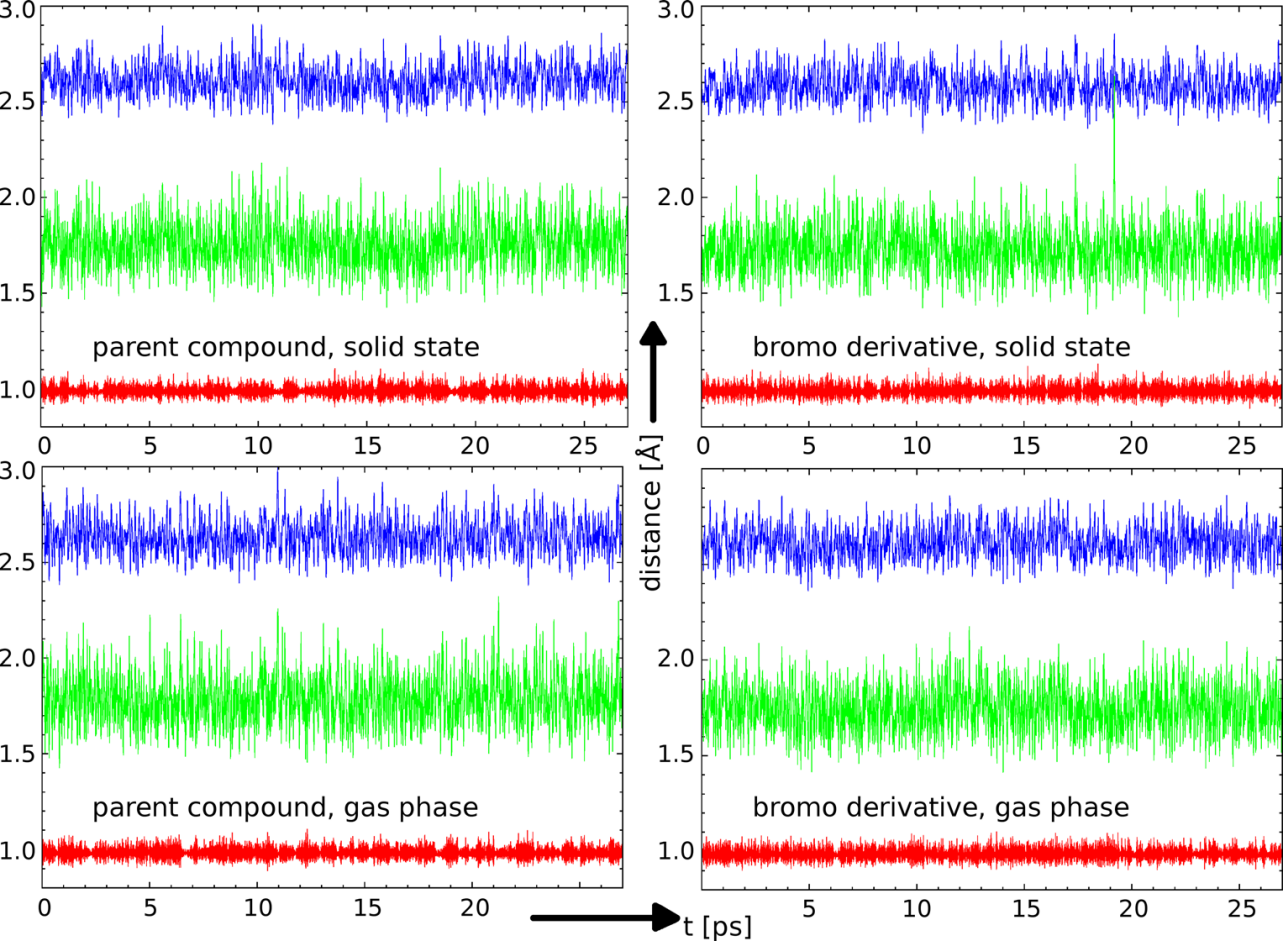
Time evolution of the interatomic distances related to the intramolecular hydrogen bond. Each graph contains the following distances: bottom (*red*): O-H, middle (*green*): H···O, top (*blue*): O···O distance. Results of the CPMD simulations

The basic structural parameters constituting the descriptors of a hydrogen bridge are donor-proton, proton-acceptor, and donor-acceptor distances. Their evolution in the time domain in the CPMD simulations of **1** and **2** is presented in Fig. 2. As shown, the phenolic proton stays at the donor site for all the investigated cases within the assumed conditions (environment, temperature, and available time scale of simulation). This fact is consistent with the difference of acidities and chemical natures of the donor (phenol) and acceptor (anisole) sites.

Another issue is related to the hydrogen bridge dynamics. The presence of a bromine atom as a substituent in the aromatic ring and isopropyl moiety in the vicinity of the intramolecular hydrogen bond in **2** is not able to change the dynamics of the interatomic distances and bond lengths of atoms involved in the hydrogen bond formation in a qualitative way (by, for example, introducing short-lived proton sharing events). The graphical presentation of the obtained time-evolution results (see Fig. 2) shows for both compounds almost identical curves. The result confirms our previous findings [7] where many electronic-structure related effects were shielded by the presence of a strong intramolecular hydrogen bond. On the other hand, the data show that from the thermodynamical and statistical points of view the inductive and steric effects of the substituents in **2** are subtle and not directly visible in the CPMD trajectory. Therefore, to get quantitative results concerning the structural impact of substitutions it is necessary to employ statistical post-processing of the trajectory. Table 1 presents selected metric parameters to show the performance of the CPMD simulations in the solid state and in the gas phase versus experimental measurements. We are aware of the approximate nature of the process of locating the hydrogen atoms (especially the bridge proton) in the X-ray measurements; we are also fully aware of the approximations (e.g., model of classical nuclei) made by us in the comparison presented in Table 1. As shown, the structural parameters involving only heavy atoms are reproduced correctly. The solid-state CPMD runs yield average values larger by no more than 0.03 Å with respect to the experiment. However, the use of gas phase results allows us to separate the substituent effects from the impact of crystal environment. It is visible that the O···O separation is larger by 0.02÷0.03 Å in the gas phase, and the solid-state shortening with respect to the isolated molecule is a combined effect of packing forces (steric hindrances) and electrostatic crystal field. However, in a chosen state of matter, the O···O distance in **2** is shorter by 0.02÷0.03 Å than in **1**. This behavior is seen in the gas phase as well, therefore it should be regarded as coming purely from the substituent effect. The *p*-Br substitution in **2** increases the nucleophilic character of the methoxy fragment making it more compatible with the naphtholic –OH donor in the sense of the Gilli’s slide rule [28]. This substitution is interesting because the bromine atom exhibits different modes of action through resonance (electron donation) and induction (electron withdrawal), as shown above in the values of the substituent constants for bromine. The interatomic O···O distance equal to *ca*. 2.6 Å indicates that the intramolecular hydrogen bond could be classified as bordering the short and strong HB region. Having this in mind, one can expect that the potential energy surface (PES) is rather flat, therefore events related to the bridge proton mobility could occur in heavily substituted analogs. The expectation is in line with design of new molecules with desired molecular features, such as proton transfer phenomena forced by, e.g., the acceptor atom exchange or by exchange of the chemical constitution, which would allow the proton transfer in agreement with classical theories of basicity and acidity of the donor-proton-acceptor subsystem [28]. Earlier, experimental [29] as well as computational [30], studies have shown that it is possible to tune the proton position in the bridge by combination of substitution and crystal field effects.

Table 1 indicates that the measured O-H bond length is too short. The CPMD simulations in the solid state and in the gas phase provide the bond length of *ca*. 0.99 Å, which is consistent with neutron diffraction findings for such bonding, e.g., ref. [6]. The hydrogen bond (H···O) was reproduced properly as well by the simulations. The CPMD average bond length of **1** is shorter than that obtained experimentally. In the case of **2** a very good agreement with experimental H···O bond length (up to 0.03 Å) was found. The experimental bond lengths suggest that in both compounds the H-bonding is middle-strong. The CPMD runs show a strong mobility of the acceptor moiety in both compounds. The quasi-ring formation stabilizes the molecular structure, but the acceptor moiety is not rigid and the part of both molecules shows movements during the MD runs. As shown in Table 1, the gas phase results show elongations of most of the interatomic distances, which is in agreement with the fact that the degrees of freedom do not have any restrictions resulting from packing. An opposite situation is found in the solid state where packing phenomena appear. Therefore, to be able to prepare a possibly accurate description of the molecules, Grimme’s dispersion corrections were included throughout the study. The only exception is the O-H bond length, shorter by 0.004 Å in the gas phase simulation of **1**, and by 0.001 Å for **2**. This delicate shortening, well below the standard deviation margins resulting from thermal motions, reflects the absence of crystal forces which compressed and shortened the bridge in the solid state, making it stronger (in which case an elongation of donor-proton distance occurs).

The O-H···O valence angle for both compounds was reproduced in a good agreement with the measured values. Its value deviates from linearity typically for the intramolecular bridges where the molecular skeleton is the deciding geometrical factor [8, 29]. The last column of Table 1 contains experimental and computed C-C-O-H torsion angle values. The purpose of the analysis was to check the planarity of the hydrogen bonding during the CPMD simulations at room temperature. The quasi-ring in the crystal structures [3, 4] is somewhat distorted in both compounds. A larger distortion is noticed for **2**, which is associated with the steric effect introduced by the presence of the isopropyl substituent. The CPMD results are in agreement with the experimental findings in the solid state. The presence of the neighboring molecules as well as the crystal field made the intramolecular hydrogen bond less able to promote planarity of the structure. An opposite situation is observed in the gas phase where both compounds exhibit more flexibility, as judged by the standard deviations of the torsion angles. This, however, cannot hide the fact that the average values of the torsion angle are in both cases close to 0, indicating that small deviations of the proton from the ring plane in the solid state result from packing forces.

### Vibrational features

**Fig. 3:**
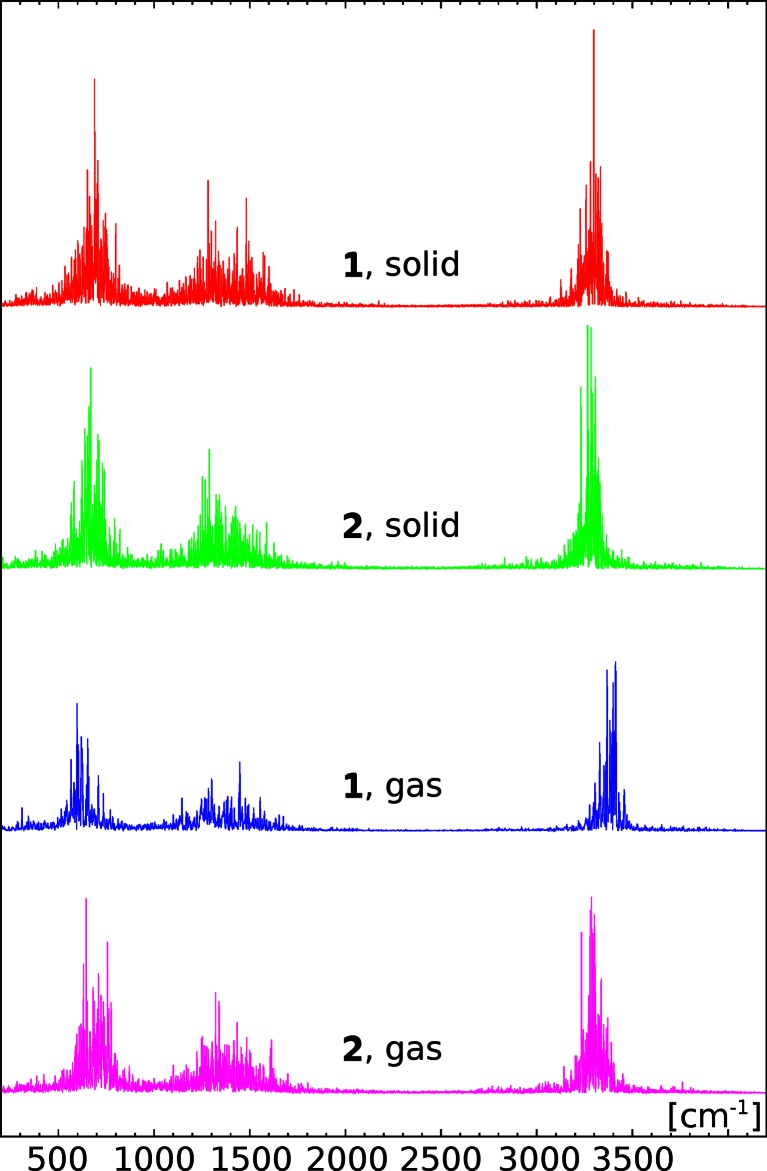
Contributions of the bridge protons to the atomic velocity power spectra. Positions are related to the vibrational features, while the intensities are arbitrary. Results of the CPMD simulations

Figure 3 presents the bridge proton contribution to the power spectra of atomic velocity. The upper part of the figure is devoted to the solid state results. The substituent effect is only minimally reflected in the spectra. For both compounds the IR signatures are basically the same. Two regions with strong intensities are detected: 300–1800 cm^−1^ and 2800–3600 cm^−1^. The first, low-wavenumber region records most of the molecular motions, since the classical-nuclei model of CPMD does not allow for ideal separation of the constituent molecular oscillators. Thus, the second, high-wavenumber region, collecting the O-H stretching coordinate, is more relevant for the study. The largest motion intensity is registered at the wavenumbers of 3300 cm^−1^ for **1** and 3250 cm^−1^ for **2**. In the case of the gas phase simulations, more diverse results are obtained. Similar to the solid state computations, two regions with strong intensities are observed. The first region is located for both compounds at 300–1800 cm^−1^. The second region is located for the parent compound at 3100–3450 cm^−1^ whereas for its derivative the region is located at 3000–3500 cm^−1^. The maxima for the O-H stretching are observed for the parent compound **1** at 3400 cm^−1^ whereas for the compound **2** at 3300 cm^−1^. As shown, the O-H stretching for **2** is shifted to lower wavenumbers comparing with those obtained for the parent compounds, which is associated with the presence of substituents. The competition between electron-withdrawing and electron-donating properties of the bromine atom (cf. the substituent constants given at the beginning of this section) located in the *para* position with respect to the hydrogen bond acceptor leads to a small increase in the proton-accepting (nucleophilic) properties of the acceptor, thus contributing to a small strengthening of the bridge. This strengthening is reflected in a small (ca. 100 cm^−1^) red shift of the corresponding O-H stretching. Concluding, the combined effect of the crystal field and the presence of intramolecular hydrogen bond was able to shield partially the substituent effects in the solid state. An opposite situation is observed for the gas phase simulations, where the computed vibrational signatures detected the influence of substituents on the molecular structure of the investigated compound, resulting in a red shift of ca. 100 cm^−1^ when moving from **1** to **2**. These results are based on atomic velocity power spectra, therefore the apparent intensities are related to motion amplitudes, not to the observable IR data. However, the spread of the stretching region (close to 500 cm^−1^ in the gas phase) is significant, indicating that the proton in the bridge is moving in a dynamically changing potential.

## Conclusions

Two naphthalene derivatives have been investigated in the current study. Knowing that the proton transfer phenomena cannot occur in these compounds, our attention was focused on geometric and spectroscopic signatures of substitution effects and the dynamics of the intramolecular hydrogen bridge. Therefore two compounds were chosen — the parent one, serving as the reference for the second one. It has been shown that for this type of compound the CPMD method is able to reproduce the structure parameters correctly, indicating that the dynamics of the hydrogen bridges was reproduced correctly as well. The employment of Grimme’s dispersion correction enabled us to include weak interactions to improve the structural model, especially in the solid state. The substituent effects seem to be subtle and they are partially shielded by the intramolecular hydrogen bond. However, they are reflected in the metric parameters, especially when comparing gas phase results — this allows for filtering out the crystal packing forces. The substituent effect is visible in the computed vibrational spectra based on Fourier transforms of atomic velocity. The O-H stretching of the substituted derivative is shifted to lower wavenumbers than those of the parent compound in the solid state as well as in the gas phase. The CPMD method provided us not only with time-evolution results for both studied compounds, but on the basis of this approach the vibrational description has been carried out and the substituent effects were highlighted.
